# Regenerative Endodontic Treatment in an Immature Permanent Tooth With Necrotic Pulp and Periradicular Lesion

**DOI:** 10.1002/ccr3.70941

**Published:** 2025-09-24

**Authors:** Mamak Adel, Zohreh Asgari

**Affiliations:** ^1^ Department of Endodontics, Dental Caries Prevention Research Center Qazvin University of Medical Sciences Qazvin Iran; ^2^ Department of Endodontics, School of Dentistry Qazvin University of Medical Sciences Qazvin Iran

**Keywords:** immature tooth, open apex, pulp revascularization, regenerative endodontics, root development

## Abstract

Managing necrotic immature permanent teeth with an open apex presents a treatment challenge. Regenerative endodontic procedures have emerged as biologically based approaches for these cases, suggesting an alternative to conventional apexification. This report describes successful revascularization of an immature mandibular second premolar with a chronic apical abscess. Following canal disinfection with NaOCl irrigation and applying a calcium hydroxide as an intracanal medicament, a scaffold was created by inducing a blood clot within the canal. During follow‐up sessions, the tooth remained asymptomatic and clinically functional. Radiographic evidence demonstrated ongoing apical root development with increases in both root length and dentinal‐wall thickness, along with signs of apical closure. The treatment outcome supports the premise that conservative pulp revascularization can promote complete root maturation by preserving the vitality of dental pulp stem cells and providing a conducive environment for pulp regeneration.

**Summary**

- In a necrotic permanent tooth with an open apex and an apical lesion, conservative pulp revascularization techniques can promote complete root maturation by preserving the vitality of dental pulp stem cells and creating an environment conducive to pulp regeneration.

## 1 Introduction

An immature apex in a tooth with pulpal damage presents a significant challenge since conventional root canal therapy is unlikely to achieve ideal obturation when the apex is not closed [1]. Consequently, treatment outcomes in these cases are variable and uncertain. Due to the apical divergence of the root canal walls in young nonvital teeth, forming a conventional apical stop is not feasible. In such situations, treatment aims to stimulate the formation of a hard‐tissue apical buttress and to prevent overfilling the canal with obturation materials [2]. Treatment options include apexification or regenerative approaches. Apexification involves creating an artificial barrier using biomaterials such as mineral trioxide aggregate (MTA) or achieving apex closure with calcium hydroxide (Ca(OH)_2_) [3, 4], but these methods do not reliably promote continued root development and often require multiple visits [5, 6, 7]. By contrast, regenerative approaches offer a biologically based alternative that allows continued root development in necrotic immature teeth [8, 9].

Current research indicates that regenerative endodontic procedures are appropriate for immature necrotic teeth with periapical lesions [10]. In immature teeth with open apices, more stem cells can migrate into the root canal system. In addition, stem cells of the apical papilla near immature root apices have a high potential for pulp regeneration [11, 12]. In clinical research, regenerative endodontic therapy appears able to strengthen immature fragile roots by increasing root length and dentinal‐wall thickness [13].

Since tissue growth halts where bacteria persist, effective disinfection of the root canal system is essential for successful regenerative endodontic treatment [14, 15, 16]. Infectious substances within the canal are eliminated through chemical irrigation and disinfection. Subsequently, mesenchymal tissue resembling dental pulp may repopulate the pulpal space [17, 18, 19]. Revascularization, which involves inducing bleeding and allowing the resulting blood clot to function as a scaffold for migrating stem cells to adhere to and proliferate, is a highly effective regenerative endodontic technique [20]. Owing to signaling molecules and bioactive calcium silicate materials, these stem cells can differentiate into dental pulp, dentin, and periodontal ligament tissues [21, 22].

Ultimately, an effective coronal seal is essential to promote continued root growth by creating an environment conducive to stem cell proliferation and differentiation. Materials such as mineral trioxide aggregate (MTA), calcium‐enriched mixture (CEM), and Biodentin can be employed for this purpose [23]. Cold ceramic has clinical applications comparable to MTA and other calcium silicate cements. It is biocompatible, non‐toxic, and possesses adequate radiopacity. Cold ceramic is used in various procedures, including root‐end filling, root perforation repair, apical barrier formation, dental pulp revascularization, and potentially as a paste for obturating root canals. It begins to set within 15 min under moist conditions, with complete setting achieved in 24 h [24, 25]. In addition to demonstrating favorable cell attachment and biocompatibility, cold ceramic has been shown to increase the expression of osteo/odontogenic differentiation markers [26]. A recent study indicates that cold ceramic achieves comparable marginal adaptation to MTA [27].

This clinical research aimed to investigate the effect of a revascularization treatment on a tooth with an open apex and an apical lesion.

## 2 Case History/Examination

**FIGURE 1:**
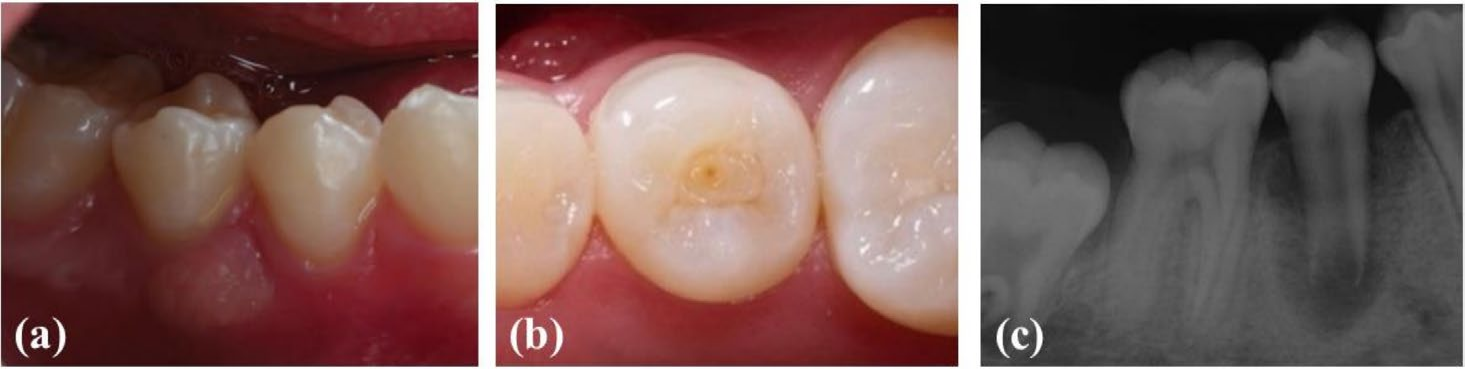
(a) A preoperative view of the mandibular right second premolar with a large localized buccally abscess, (b) intraoral photograph of tooth showing a small tubercle on the occlusal surface, (c) a preoperative periapical radiograph of tooth with immature root and large periradicular lesion.

An 11‐year‐old female patient was referred by a general dentist to the endodontic clinic at the School of Dentistry, Qazvin University of Medical Sciences. The chief complaint was pain during mastication, and the patient reported a two‐week history of an abscess on the right mandibular posterior tooth. The patient had not previously treated the injured tooth. There was no significant medical history or known drug allergies. An extraoral examination revealed no abnormalities. The head and neck examination showed no palpable lymphadenopathy. According to the intraoral examination, the patient demonstrated regular oral hygiene, though an abscess was localized buccally to the right mandibular second premolar (Figure 1a). Clinical examination revealed a small tubercle on the occlusal surface of the tooth, while the molars and premolars were free of caries (Figure 1b). The affected tooth showed no discoloration. Periodontal probing and physiological mobility were within normal limits. Diagnostic tests with cold, hot, and electric pulp tests were inconclusive, with only mild percussion sensitivity. Radiographic examination demonstrated a large periradicular lesion associated with an immature root with a wide open apex, and root development appeared arrested (Figure 1c).

## 3 Diagnosis and Treatment

Based on the patient's history, clinical examination, and radiographic imaging, the initial diagnosis for the right mandibular second premolar was pulpal necrosis with a chronic periapical abscess. Various treatment options were discussed with the patient, including the apical barrier technique and pulp revascularization. After thoroughly explaining the advantages and disadvantages of both approaches, the patient elected pulp revascularization, and informed consent was obtained.

At the first appointment, local anesthesia was administered via an inferior alveolar nerve block using 2% lidocaine with 1:80,000 epinephrine (DarouPakhsh, Tehran, Iran). The mouth was rinsed with 0.2% chlorhexidine gluconate, and the tooth was isolated with a rubber dam. A typical access cavity was prepared with a high‐speed handpiece and a medium round diamond bur. The pulp chamber released a purulent and bloody fluid, and the pulp's necrotic state was affirmed. The working length was determined using an electronic apex locator (Root ZX II, Morita, USA) and confirmed by radiography. The root canal was irrigated adequately with 20 mL of 1.5% NaOCl for 5 min without mechanical instrumentation. The irrigating needle (#30‐gauge, side‐vented) was positioned approximately 1 mm from the root end during the process. To preserve progenitor cells and avoid damaging the thin dentinal walls in the apical tissues, NaOCl was delivered using passive irrigation [28]. A final rinse with 20 mL of normal saline was performed for 5 min to remove residual NaOCl. A creamy Ca(OH)_2_ paste (EX Cidox, Nikdarman, Iran) was placed into the canal using a lentulo spiral (Mani, Japan) after drying the canal with sterile paper points. Finally, a temporary sealing cement (3M Espe, Seefeld, Germany) was placed in the access cavity, and the patient was scheduled for a follow‐up visit in three weeks later.

**FIGURE 2:**
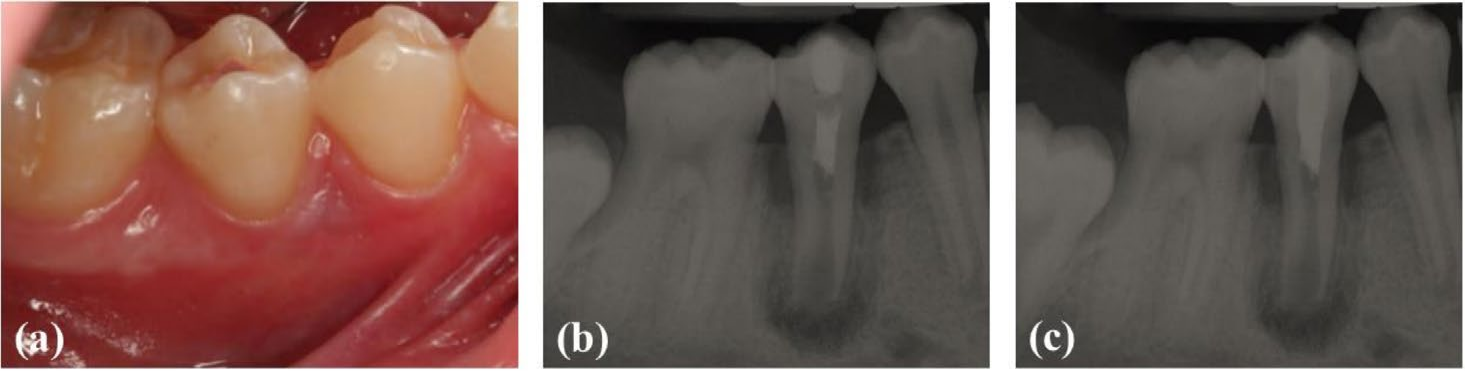
(a) Subsidence of buccal gingival swelling, (b) confirming radiograph the placement of cold ceramic, (c) post operative radiograph of restored tooth.

At the second visit, buccal gingival swelling had resolved (Figure 2a), and the tooth was asymptomatic. Local anesthesia was achieved with an inferior alveolar nerve block using 3% plain mepivacaine (DarouPakhsh, Tehran, Iran), and the tooth was isolated with a rubber dam. The temporary sealing material was removed. Root canal irrigation was performed with 30 mL of 17% ethylene diamine tetraacetic acid (EDTA) solution (Asia Chimi Teb Co.) for 5 min, followed by ultrasonic activation. A final irrigation with 5 mL of normal saline for 1 min was performed, the root canal dried with sterile paper points, and the absence of exudate was confirmed. To induce bleeding into the root canal space, a #25 K‐file (Mani Inc., Tochigi, Japan) with a slight bend at the tip was applied 2 mm beyond the apical foramen to lacerate the periapical tissues. Approximately 3 mm below the cementoenamel junction (CEJ), bleeding was allowed to form a clot for 15 min. The clot was then covered with 3 mm of cold ceramic (Monsefteb, Yazd, Iran), and a moist sterile cotton pellet was placed atop the cold ceramic. Temporary sealing cement was used to seal the access cavity. Subsequently, a radiograph was taken to confirm the placement of the cold ceramic (Figure 2b).

The patient returned 48 h later, remaining symptom‐free. The cotton pellet and temporary restorative material were removed from the access cavity, and the complete setting of the cold ceramic was verified. The affected tooth was restored with a light‐cured composite resin (3M ESPE, Filtek Z250 XP, USA), and a periapical radiograph was obtained (Figure 2c). The patient was scheduled for a follow‐up visit and advised to contact the clinic if pain, swelling, or an abscess recurrence occurred.

## 4 Conclusion and Results

**FIGURE 3:**
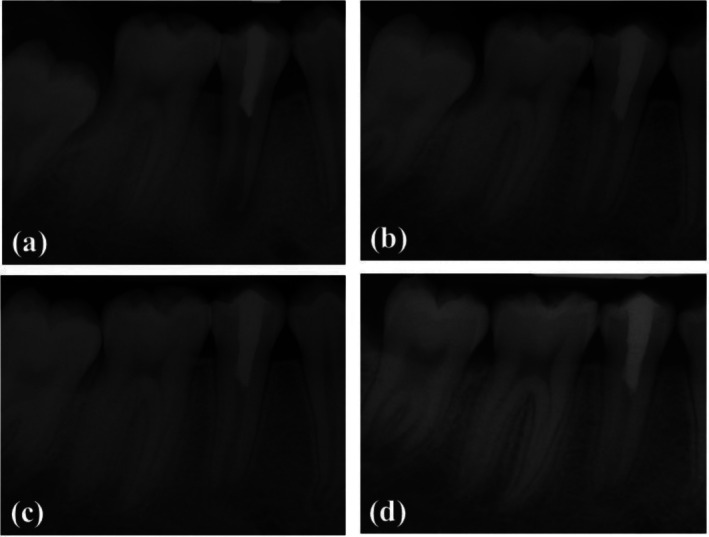
Follow‐up preapical radiographs. (a) 1‐month follow‐up, Radiographic signs of healing periradicular lesion; (b) 6‐month follow‐up, complete disappearance of periradicular radiolucency; (c) 1‐year follow‐up, display of continued root development; (d) 2‐year follow‐up, display of complete root development.

At the 1‐month follow‐up, the patient reported no symptoms since treatment. The tooth was not sensitive to percussion and palpation. The periradicular radiolucency had diminished considerably (Figure 3a). At the 6‐month recall, the patient remained symptom‐free with no signs of abscess formation, and the periradicular radiolucency had resolved completely (Figure 3b). By the 1‐year follow‐up, the patient continued to be symptom‐free and without signs of abscess. The periodontal ligament was thin, and the periradicular radiolucency zone had entirely healed, a sign of the tooth apex continuing to develop (Figure 3c), whereas the results of pulp testing were still inconclusive. At the 2‐year follow‐up, the patient remained asymptomatic, and it was clear that the dentinal walls had thickened and the apex had closed (Figure 3d). Cold and EPT testing were positive for the tooth (Table 1 summarizes the outcomes). Compared with the apical barrier approach, pulp revascularization showed a favorable outcome in promoting root development and could be considered a reliable alternative to conventional apexification in carefully selected cases.

**TABLE 1:** Pulpal and periapical tests in pre‐treatment and follow‐up sessions.

Time	Tests					
	Cold	Heat	EPT	Percussion	Palpation	Description
Pre‐treatment	−	−	−	+	Normal	A large periradicular lesion, and an immature root with a wide open apex
1‐month follow‐up	−	−	−	Normal	Normal	Signs of healing periradicular lesion
6‐month follow‐up	−	−	−	Normal	Normal	Complete disappearance of periradicular radiolucency
12‐monrth follow‐up	−	−	−	Normal	Normal	Root development continued
24‐month follow‐up	+	−	+	Normal	Normal	Thickening dentinal walls and closing apex Root development completion

## 5 Discussion

Because the revascularization approach promotes thicker and longer root development, resulting in a tooth that is less prone to fracture, several studies have described it as an efficient alternative treatment [29, 30, 31]. Currently, no accepted criterion exists for assessing the effectiveness of pulp revascularization. Clinical appearance, pulp vitality testing, and radiographic examination have been the primary factors determining the treatment outcomes. According to the AAE guidelines, the main objectives include healing of apical periodontitis and resolution of clinical symptoms. Although a positive pulp vitality response, increased dentinal‐wall thickness, and continued root formation are favorable indicators, they are not required for success [32]. In the presented case, all treatment aims were achieved, including resolution of clinical infection, continued root development, and a positive pulp vitality response.

For successful pulp regeneration, revascularization requires three essential steps: disinfection of the intracanal space, the administration of a scaffold to organize host stem cells, and a robust coronal seal [33]. These factors are necessary for the generation of functional tissue [34]. In the present case, to preserve a larger population of viable stem cells, a low concentration of NaOCl (1.5%) was used for canal disinfection and removal of necrotic tissue, in contrast to several earlier case reports [35, 36, 37, 38]. Martin et al. reported that 1.5% NaOCl enhanced dentin sialo phosphor protein (DSPP) expression in stem cells, whereas 6% NaOCl markedly decreased the survival and odontogenic differentiation of apical papilla stem cells [39]. Following AAE recommendations, the final irrigation solution was EDTA [40]. The chelating action of EDTA facilitates the release of growth factors entrapped in dentin, thereby promoting stem cell proliferation [41]. Intracanal medications such as triple antibiotic paste (TAP) or Ca(OH)_2_ are commonly advised after disinfection. Due to the disadvantages of TAP, involving tooth discoloration and relatively high cost, it was not used in this case [42]. Ca(OH)_2_ was applied as an intracanal medicament to disinfect the canals between appointments; this is considered critical for regenerative endodontic procedures [43, 44]. In contrast to antibiotics, using Ca(OH)_2_ for disinfection preserves the viability of stem cells in the apical papilla (SCAP) [45], and Ca(OH)_2_ leads to more apical closure than TAP in some analyses [46].

In revascularization procedures, clinicians apply tissue engineering principles to promote pulp tissue regeneration and continued root apex maturation. Numerous case reports indicate that inducing bleeding from periapical tissues into the root canal can serve as a source of progenitor cells [47, 48]. However, intra‐canal bleeding can be variable, and periapical tissue injury may result in little or no blood. Nevertheless, the blood clot that forms within the canal serves as a scaffold to support new tissue formation [49, 50, 51]. Whole blood clots perform superior in terms of root elongation, thickness, and sensitivity test responsiveness compared to other scaffolds like platelet‐rich fibrin (PRF) and platelet‐rich plasma (PRP) in terms of root elongation, thickness, and sensitivity test responsiveness [52].

Because hydraulic calcium silicate cements can set in the presence of moisture, they are recommended for covering the blood clot to achieve a hermetic coronal seal [53]. In this study, we used cold ceramic as the preferred alternative calcium silicate cement for the coronal seal, given its favorable handling properties, consistent mix, non‐toxicity, and biocompatibility with host tissues [54]. Study results indicate that the sealing ability of cold ceramic surpasses that of MTA in blood‐contaminated environments; however, in dry and saliva‐contaminated conditions, cold ceramic is comparable to MTA. MTA exhibits a setting time of approximately 165 min, whereas cold ceramic sets more rapidly, at about 15 min. Additionally, there was no discernible difference in tooth discoloration between MTA and cold ceramic [24]. Hydraulic calcium silicate cement should be placed cautiously just below the CEJ to minimize the risk of discoloration and other unfavorable effects [41].

The origin of the newly formed pulp tissue is a fascinating subject. While we must acknowledge uncertainty about whether the regenerating tissue is truly pulp, the tissue in this case was most likely pulp with active odontoblasts, as evidenced by continued root growth and conventional thickening of the root walls. Although the majority of the pulp in immature teeth is typically infected and devitalized, it is possible that some pulp tissue survived apically. Consequently, a remnants of vital pulp tissue and Hertwig's epithelial root sheath may have persisted despite a notable apical lesion. Proliferation of these tissues can occur once the canal is disinfected and the inflammatory milieu is addressed [55, 56].

Significant improvements in clinical and radiographic characteristics were observed throughout the follow‐up, confirming successful postoperative outcomes. The patient remained symptom‐free, with evidence of continued root development and apical closure. In this case, the effectiveness of the regenerative endodontic procedure was confirmed by follow‐up visits at 1, 6, 12, and 24 months post‐treatment, which demonstrated ongoing healing and regeneration.

## Author Contributions

**Mamak Adel:** project administration, resources, validation, visualization, writing – review and editing. **Zohreh Asgari:** conceptualization, data curation, investigation, methodology, resources, writing – original draft.

## Ethics Statement

This case report meets the ethical guidelines and adheres to Iran's local legal requirements.

## Consent

Written informed consent was obtained from the patient to publish this report in accordance with the journal's patient consent policy.

## Conflicts of Interest

The authors declare no conflicts of interest.

## Data Availability

The data that support the findings of this study are available on request from the corresponding author. The data are not publicly available due to privacy or ethical restrictions.
